# Dr. Barry's Reply to the Letter of Mr. Fraser, in Our Last Number

**Published:** 1831-02

**Authors:** 


### DR. BARRY AND MR. FRASER.

### Dr. Barry's *Reply to the Letter of* Mr. Fraser, *in our last Number.*

*To the Editor of the London Medical and Physical Journal.*

Sir: In your Journal published this day, there is a letter signed "Hugh Fraser," in which the author calls upon me to publish my paper on the Gibraltar Epidemic, read last spring, at the Royal College of Physicians, or to lodge a copy "in your hands, so that he may have access to it."

May I beg that you will have the kindness to inform your correspondent, that I deposited a copy of that paper more than six weeks ago, with Dr. Gordon, in Berkeley street, for the perusal of any one who might think it worth that trouble. The original being the property of the College, I have this day called on the registrar, who has kindly promised to forward the paper to you immediately, and also to apply to the committee of the College for permission to have it published. Should that permission be obtained, you will oblige me much by inserting it in an early number of your Journal, if you deem it worthy of the honour.*

In that imperfect, elementary sketch, (necessarily short, that it might be read within an allotted number of minutes,) I think you will perceive, that I have carefully respected both persons and opinions; that I have quoted Mr. Fraser as leading authority, and that I have not even alluded to one of the points, in which he seems to have imagined my very faint outline of the epidemic as the most unfaithful.

Permit me, sir, to protest against the gratuitous introduction of Sir William Pym's name, as in any way connected with my defensive reply to Mr. Fraser's attack. The faults of that reply, numerous as I freely allow them to be, must all rest at my own door exclusively. Sir William was out of town when it was written, nor did he return before it was printed. His disapproval of its style, when he did see it for the first time, would at once have induced me to reject, had it not been too late, the irony and badinage which, it is evident, have not been taken in the good-humour they were intended, if possible, to preserve.

My medical brethren of Gibraltar, and all those who at the time took an interest in the history of the epidemic, from whom I may have the misfortune to differ in opinion on that subject, will, I hope, make large allowances for the feelings of a man attacked as I have been. To many of these gentlemen I am largely indebted for repeated kindnesses and hospitality; to all, for the obliging candor with which they have met my inquiries. To have bestowed praise on the medical corps of Gibraltar, would be presumption and egotism in me, who had the honour of belonging to it for more than a year. Many of its members were my seniors in the service, and almost all of them, in personal experience of the disease. They have had both praise and honours from the very highest sources, from which neither would have come, had they not been deserved. To have talked slightingly of them, would have been to vituperate myself. I have, therefore, abstained alike from compliment and criticism: certain it is, at least, that neither was intended.

After this avowal of feelings, which must still prevent me from giving utterance to the eulogies their devotedness so nobly earned, I trust that my learned confrères of Gibraltar will not permit a harsh interpretation of a harmless, local allegory, nor an attempt at humor, however unsuccessful, to rob

* The paper to which Dr. Barry refers forms the first article of this number of our Journal, and was received direct from the registrar of the College of Physicians, at Dr. B.'s request.—Editor.

me of whatever portion of their esteem I may have previously gained. The insipidity and utter uselessness of purely personal polemics would, I was afraid, have rendered my production unreadable, without a little raillery. Henceforward, however, I shall try to be dull and dignified, even in opposition to the interrogatory of the witty Roman satirist:

"Quid vetat ridentem dicere verum?"

Mr. Fraser having, early in this controversy, "disavowed all personal animosity or hostile feeling towards me," I should, in courtesy, have echoed this liberal declaration in my reply. In order, however, to make amends for the omission, I now declare, that he has held a high and friendly place in my estimation ever since I have known him; and even at this moment I think him incapable of any motive towards me, incompatible with an honourable mind. From my soul I believe, that the seemingly contradictory assertions, into which he appears to have been hurried by an over-jealous zeal for his own and his friend's opinions, are owing, as I have hinted in my reply, to his having entirely forgotten the existence of some documents, and to not having carefully examined, or perhaps not been at all acquainted with others.

Mr. F., whatever may be his views as to the communicability of yellow fever, must allow that polemic acrimony of language is highly contagious, particularly in the predisposed. As I have had *one* slight attack of the latter disease, I shall, of course, continue to be exempt from it in future; and Mr. F. being, as I think you will *now* admit, a well-marked case of double attack of this verbal malady, will not, it is to be hoped, consider a *third* paroxysm necessary in his search after truth, however portentous the disclosures which its acquisition is intended to produce.

I remain, sir, your very obedient servant,

D. BARRY.

*London; January* 1*st*, 1830.

P.S. Allow me to recall your attention for a moment to Mr. Fraser's letter already described, but more particularly to the manuscript from which it was printed, and which, at my desire, you have permitted me to examine. On that manuscript I must take the liberty to remark:

That, well aware as I am, from what I know of Mr. Fraser's talents, that he needs no assistance in preparing his papers for the press, I could not help being forcibly struck with certain interlineations and additions, not of the most courteous kind, penned in a hand-writing widely different from that in which the body of the letter is written (Mr. Fraser's), with which I happen to be well acquainted.

Amongst these additions of new matter, the following are observable:

1st. The most important word in the uncivil note at page 32 of your last number, referring to a third person, and implying a serious impeachment of others.

2d. A whole extraneous paragraph, (page 33,) wafered on to the margin of the manuscript, with marks directing the printer where to insert it in the text.

Now, sir, under the fullest conviction of the even-handed justice with which you hold the controversial balance, and without envying Mr. Fraser the entire authorship (should he claim the undivided honour,) of that, or any other such paper, wholly or partially written by a hand not his own, I must yet be allowed to complain that you should have published a paper purporting to come from Mr. Fraser, of which the most severe and offensive parts were evidently not in his hand-writing.*

* Our answer to Dr. Barry's complaint, (at which, in truth, we are not much astonished,) may be included in a few words. The manuscript of Mr. Fraser's letter was placed in our hands by Dr. Gillkrest. The last page of that manuscript, signed by Mr. Fraser, had many erasures, interlineations, and material alterations, in a different hand-writing. Of this last page Dr. Gillkrest gave us a fair copy, which he assured us was a correct transcription of the original. It was, accordingly, sent to the press instead of the page of the original referred to. The following letter from Mr. Fraser shows that he authorised the insertion of the various alterations which Dr. Barry mentions. Two words we took upon ourselves to strike our pen through in Mr. Fraser's original manuscript: it appeared to us that they were unnecessarily severe, and not called for by any thing which Dr. Barry had published in our Journal.—Editor.

*Chatham*, 15*th January*, 1831.

Sir: In compliance with your desire on the subject, I beg to state, that the letter placed in your hands last month for publication, was an exact copy, although not in the same hand-writing throughout, of one which I had made out and forwarded from this; and which I finally determined to be the whole of what I should then say, as time did not permit of my entering into details connected with the controversy in which I am now engaged.

I have the honour to be, sir, your most obedient, humble servant,

HUGH FRASER.

To John North, Esq.
Editor of the Med. and Phys. Journal.

